# Episodic Autobiographical Memory Impairment and Differences in Pronoun Use: Study of Self-Awareness in Functional Amnesia and Transient Global Amnesia

**DOI:** 10.3389/fpsyg.2021.624010

**Published:** 2021-10-14

**Authors:** Céline Becquet, Julien Cogez, Jacques Dayan, Pierrick Lebain, Fausto Viader, Francis Eustache, Peggy Quinette

**Affiliations:** ^1^“Neuropsychology and Imaging of Human Memory” Research Unit, Caen-Normandy University-PSL Research University-EPHE-INSERM-Caen University Hospital, Caen, France; ^2^Neurology Department, Caen-Normandy University Hospital, Caen, France; ^3^Pôle Hospitalo-Universitaire de Psychiatrie de I’Enfant et de l’Adolescent, Centre Hospitalier Guillaume Régnier, Université Rennes 1, Rennes, France; ^4^Psychiatry Department, Caen-Normandy University Hospital, Caen, France

**Keywords:** self-awareness, I-self, episodic autobiographical memory, narratives, transient global amnesia, functional amnesia, dissociation

## Abstract

The subjective experience associated to memory processing is the core of the definition of episodic autobiographical memory (EAM). However, while it is widely known that amnesia affects the content of memories, few studies focused on the consequences of an impairment of EAM on the subjective self, also called the *I-self*. In the present study, we explored the I-self in two puzzling disorders that affect EAM: *functional amnesia*, which has an impact on autobiographical memory, and *transient global amnesia* (TGA), which only affects episodic memory. I-self was assessed through an original measure of self-integration in autobiographical narratives, namely the use of general or personal pronouns. Results showed that patients with functional amnesia tended to use general pronouns, whereas patients with TGA preferentially used the first person. The link between *I-self* and depersonalization-derealisation tendencies was also explored, showing dissociative tendencies in patients with functional amnesia but not in patients with TGA. We discuss these results from a combined neuropsychological and psychopathological perspective, with a view to proposing an explanatory model of the links between self-awareness and the episodic component of autobiographical memory.

## Introduction

*Episodic autobiographical memory* (EAM) (Conway et al., 2004) is located at the junction of two concepts: *autobiographical memory* (AM) and *episodic memory*. The former describes a theoretical memory construct that stores all the memories and information supporting identity (Conway and Pleydell-Pierce, 2000). Thus, AM includes memories of past events but also projections into the future and interacts dynamically with the present motivations and purposes of the subject. It is composed by specific-event memories (events having happened once, of less than 24 h duration and spatially and temporally located) and general information about the self and temporally extended events or lifetime periods, sometimes named *semantic autobiographical memory* (Conway and Pleydell-Pierce, 2000; Martinelli et al., 2013). On the other hand, episodic memory allows individuals to encode, store and retrieve specific events with a subjective feeling of owning these memories and to re-experience them (Tulving, 1985; Tulving et al., 2004). This phenomenon was compared to a subjective mental travel in time. At their junction, EAM can be defined as a system involved in the process of personal or autobiographical specific-event memories, at all stages of memory processing, e.g., encoding a new relevant autobiographical event, retrieve one from the past or provide a personal specific projection in the future, by integrating the subjective dimensions described in the definition of episodic memory (Conway et al., 2004).

The basic psychological processes allowing individuals to be aware of their own involvement in subjective experiences are largely unknown. The *I-self*, also called the *minimal* or *core self*, refers to the most basic relationship between individuals and themselves when the content of their consciousness is oriented toward the environment. It subtends the sense of agency and the sense of ownership that enable individuals to feel that they are the agents of their acts and possess their own bodies (Gallagher, 2000). It could be involved in the EAM, supporting the subjective feeling of owning the memory, named by Tulving (1985) *autonoetic consciousness* (Prebble et al., 2013). In clinical populations, such as in schizophrenia, the idea of a link between I-self impairment and the phenomenological dimension in EAM has sometimes been evoked (Berna et al., 2017). In healthy individuals, an experimental study looking into the encoding of new self-relevant information showed that experimentally induced I-self impairment (out-of-body illusion) can have negative effects on the volume of EAM encoding (Bergouignan et al., 2014). In the same way, in an EAM paradigm using virtual reality, Bréchet et al. (2019, 2020) showed that first-person view of one’s body during encoding and retrieval sessions increased retrieval abilities in EAM compared to out-of-body condition. The reverse relationship between EAM and I-self functioning has not so far been explored, especially in amnesic patients.

Parallels can be drawn between the alteration of the I-self and the psychiatric concept of dissociation (Steele et al., 2009). *Dissociation* is defined as a disturbance of the normal integration of consciousness, memory, identity, emotion, perception, body representation, motor control, and behavior (American Psychiatric Association, 2013). It is sometimes described as a defensive mechanism of collapse that can be triggered by an intense stressor when fight and flight are both impossible (McKinnon et al., 2016). This mechanism can persist well after the objective threat has disappeared, to the point where it becomes the usual way of processing information. Among the symptoms described under the denomination of dissociative symptoms, especially *depersonalization-derealization* symptoms are close to the idea of I-self impairment (Holmes et al., 2005; Steele et al., 2009). They are characterized by impaired integration of information and communication with the world (derealization) and/or the individual’s own body (depersonalization). They are often described in psychiatric disorders such as PTSD, major depression disorder, schizophrenia, and borderline personality disorder (Allen et al., 1999).

A negative impact of these symptoms has been observed on the EAM system, especially on the encoding of new information (Steele et al., 2009; McKinnon et al., 2016). Rivera-Vélez et al. (2014) showed that high levels of dissociation are correlated with impaired performance on memory tasks featuring verbal and visual indices in adults with PTSD. Experimental studies where derealization symptoms are enhanced using mirror-gazing paradigms (Caputo, 2010a,b) have demonstrated a reduction in verbal (Brewin et al., 2013) or visual (Brewin and Mersaditabari, 2013) material encoding, suggesting a link between depersonalization-derealization and the encoding of new information.

In the present study, we tried to learn if impairments in EAM can impair the integration of the self as a subject (the I-self) into narratives of past memories and if there is a link between the I-self involvement and dissociative depersonalization-derealization tendencies. We tested these hypotheses on two populations presenting impairment of EAM from different etiologies: patients with functional amnesia (FA) and patients with transient global amnesia (TGA).

*Functional amnesia* (FA), or *dissociative amnesia*^1^ is classically described as a disproportionate disorder of AM in the absence of damage to the neural pathways of memory (De Renzi et al., 1997; Staniloiu and Markowitsch, 2014). Because of the peculiar neuropsychological profile of patients with FA, this condition is sometimes called *focal retrograde amnesia* (FRA), or *disproportionate FRA* (Piolino et al., 2005). Patients are often described as having massive retrograde memory disorders with no clear organic or psychogenic etiology (Staniloiu and Markowitsch, 2014). Nevertheless, rare cases of anterograde functional amnesia are documented. In these cases, patients are unable to form new autobiographical memories (Staniloiu et al., 2018). Both the semantic and episodic components of AM can be disrupted, sometimes leading to a complete loss of identity (Staniloiu et al., 2010; Markowitsch and Staniloiu, 2011). By contrast, preserved non-personal retrograde memory is sometimes observed in FA patients (Kritchevsky et al., 2004; Hennig-Fast et al., 2008). Except for the rare cases evoked above, the anterograde amnesia is generally less severe than the retrograde amnesia (Staniloiu et al., 2018), supporting the conception of FA as an *amnesic block syndrome* (Markowitsch, 2002; Staniloiu and Markowitsch, 2014). Moreover, when patients are able to recall some memories despite the *blockade*, these memories are described as lacking the subjective feeling of self, and do not seem to correspond to genuine mental time travel (Staniloiu et al., 2010; Arzy et al., 2011). This disorder can be interpreted as an impairment of the episodic autobiographical memory, and may be related to a kind of I-self alteration. Furthermore, the blockade or inaccessibility of some parts of memory or consciousness constitutes another dimension of dissociative symptoms, called *dissociative amnesia* or *structural dissociation*, which suggests a relationship between functional amnesia and dissociative processes (Bernstein and Putnam, 1986; Steele et al., 2009).

On the other hand, transient global amnesia (TGA) is classically described as a transient organic amnesia that purely affects episodic memory, with preservation of other cognitive functions. This syndrome is characterized by a sudden disruption of episodic memory associated with iterative questioning and temporal disorientation, which lasts less than 24 h and results in a full recovery. Patients with TGA have a profound disorder of anterograde and retrograde episodic memory, with an inability to recall specific memories during the acute phase that gradually improves during the recovery phase (Guillery-Girard et al., 2004). Beyond the purity of the episodic memory dysfunction, the transient nature and short duration of the disturbance are such that patients do not compensate for their deficit as they do in permanent amnesias, making TGA a valuable model for studying episodic memory. Beyond its usefulness for constructing episodic memory models, TGA is a puzzling amnesia that has several similarities with FA (Kritchevsky et al., 1997; Harrison et al., 2017). Although MRI scans have revealed delayed hyperintensities in the hippocampus (CA1), TGA is not a consequence of traumatic lesions to organic structures, and its physiopathological origin is still unknown. Like FA, TGA seems to have a psychogenic dimension, although it is not systematically found (Noël et al., 2015). One of these psychogenic dimensions concerns the possible presence of psychological precipitating events. These events may be either psychological or physical, and consist of an abrupt change in physical activity, temperature or the atmospheric environment, or else an emotional autonomic bodily response. In many cases, a general context of *psychological pressure* linked to personal (family, professional, etc.) problems is reported prior to the occurrence of the episode. Contrary to patients with FA, patients with TGA do not display a loss of identity (patients are able to answer questions about themselves). Self-awareness can nevertheless be altered in this population, resulting in patients’ inability to realize their current memory loss status during the acute phase (Hainselin et al., 2012). Given these patients’ profile namely pure episodic impairment combined with self-awareness changes but without dissociative symptoms (Guillery et al., 2001), their narratives of past memories may be related to an I-self impairment.

The present study was therefore designed to find indicators of I-self functioning in EAM retrieval in these two groups of patients (FA and TGA) presenting EAM disorders with different etiologies. Patients with FA and TGA are assumed to have an episodic autobiographical impairment, which are associated with dissociative symptoms in FA patients. We therefore undertook a comprehensive assessment of EAM encompassing three dimensions of episodic processing, namely richness (internal details), feeling of re-experiencing, and spatiotemporal specificity (see “Materials and Methods” section). We expected the two groups to exhibit comparable profiles of EAM alteration.

Our main question was whether alteration of the episodic autobiographical memory can be related to I-self impairment. We chose to avoid the use of self-report methodologies to study self-awareness disturbances. Instead, we directly examined autobiographical narratives for quantitative indicators of I-self disturbance (pronoun use). According to Gallagher (2000) who evoked the philosophical “*immunity principle*,” when an individual uses the first-person in reference to his/herself, he/she is immunized from making a mistake about the person to whom he/she is referring. Then, the use of the first-person into a narrative could be an indicator of the awareness of this individual to be the owner and the agent of his/her narrative. Here, we assumed that participants without I-self disturbances would place themselves in their narratives as the protagonists. This *self-integration* would be quantitatively reflected in the use of first-person pronouns, rather than general pronouns. We also explored the dissociative profiles of our participants on a dissociative scale, focusing on the subscale assessing depersonalization tendencies (presumably related to I-self functioning).

Then, we expected the FA group to display more dissociative amnesia symptoms than either the TGA group or healthy controls. Given the ambivalent literature about links between I-self and EAM functioning in amnesic syndromes, we put two different hypotheses. First, if there is a link between EAM disorder and I-self impairment, we would observe the same indicators of I-self disturbance (reduction in first-person use and increase in general pronoun use) in patients with TGA as in patients with FA, compared with healthy controls. Second, if there are links between dissociation and I-self impairment, but no link with memory functioning, we would find I-self impairment solely in patients with FA. In this case, patients with TGA would be equivalent to healthy controls. We then checked the hypothesis of a link between I-self and dissociative profiles, especially between use of the first-person pronoun and depersonalization-derealization tendencies.

## Materials and Methods

### Participants

This study was conducted with 10 patients (5 with FA and 5 with TGA). The two patient groups differed on age (*U* = 0, *p* < 0.05), but not education level (*U* = 12, *p* = 1). Two groups of healthy control participants (FA controls and TGA controls) were formed to match each group of patients on age and education level. Anterograde memory scores were calculated from the strategic learning component of a verbal episodic memory task measuring encoding, storage and retrieval (ESR-r; Eustache et al., 2015). This task consists of the verbal learning of a list of words involving the strategic processing of information (formulation of 16 sentences containing these words). We used the immediate free recall score (/16), expressed as *z* scores in Tables 1, 2. This methodology was used for all participants except for one patient with FA (HS; see description below), who was given a non-verbal test (TNI-93; Dessi et al., 2009). All patients gave their oral informed consent to the use of their neuropsychological data for research purposes.

**TABLE 1:** Characteristics of patients with functional amnesia (FA).

	**MC**	**CG**	**HS**	**CC**	**LA**	**Mean (range) for patients with FA (*n* = 5)**	**Mean (range) for FA controls (*n* = 8)**	***p***
Gender	F	F	M	M	M	(2F/3M)	(4F/4M)	
Age	26	24	43	42	40	35 (24–43) ± 9.22	40.1 (23–58) ± 16.05	0.71
Occupation (*years of education*)	Physiotherapist (17)	Hairdresser (11)	Factory worker (9)	Warehouse clerk (11)	Roofer (11)	11.8 (9–17) ± 3.03	12.8 (11–17) ± 2.31	0.38
Possible precipitating event	Witnessed accident at 6 years	Transient ischemic attack	Transient ischemic attack anniversary	General anesthesia	Loss of consciousness of indeterminate origin			
Duration of amnesia	20 years	10 months	1 year, 2 months	2 years	6 months			
Anterograde memory score ^‡^ (*z* score)	0.35	−0.67	−3.85**	−0.21	−1.58^†^	8.4 ± 2.79	10.5 ± 2.20	0.14
DES scores (%)								
Total	34.89	22.5	19.29	33.57	21.96	26.4^♢^	7.77	<0.01**
Dissociative amnesia	37.5	18.57	29.29	33.57	22.86	± 7.23	± 6.04	
Depersonalization-derealization	25.95	6.5	5.5	16.5	10			

^‡^ESR-r strategic free recall score or TNI 93 free recall (only for HS). Group comparisons were performed with Mann-Whitney non-parametric tests. Individual z scores were compared with normative data from Eustache et al. (2015) for ESR and from Dessi et al. (2009) TNI 93 for Patient HS. *p < 0.05. **p < 0.01. ^†^p = 0.06.

*^♢^DES total score (mean ± SD).*

**TABLE 2:** Characteristics of patients with transient global amnesia (TGA).

	**MP^2^**	**TC^2^**	**CP^2^**	AD^*^^2^	GC^*^^2^	**TGA group (min-max ± *SD*)**	**TGA control group (min-max ± *SD*)**	***p* ^‡^**
Gender	F	F	F	F	F	5 (5F)	6 (2M/4F)	
Age	75	48	62	68	66	63.8 (48–75) ± 10	63.5 (60–70) ± 3.94	0.52
Occupation (*years of education*)	Retired secretary (9)	Teacher (17)	Nurse (15)	Retired store-keeper (8)	Retired English teacher (16)	13 (8–17) ± 4.18	10.7 (9–12) ± 1.03	0.58
Possible precipitating event	Extended context of stress	Overwork	Endoscopy	Extended context of stress	Painful constipation			
Duration of amnesia	5 h	4.5 h	3 h	7.5 h	8.5 h			
Anterograde memory score (*z* score)	−3.12**	−3.85**	−5.24**	−3.12**	−4.44**	0.4 (0–2) ± 0.89	8.33 (4–11) ± 2.86	<0.01**
DES scores (%)								
Total	3.21	8.21	21.07	13.11	16.96	12.5^♢^	10.8	0.41
Dissociative amnesia	0	7.14	20	20	12.14	± 7.04	± 11.5	
Depersonalization-derealization	5	0	6	7	7.5			

ESR-r strategic free recall score. ^‡^Group comparisons were performed with Mann-Whitney non-parametric tests. Individual z scores were compared with normative data from Eustache et al. (2015). *p < 0.05. **p < 0.01.

*^♢^DES total score (mean ± SD).*

#### Patients With Functional Amnesia

Neuropsychological testing of the patients with FA was carried out during a neurological outpatient consultation at the Neurology Department of Caen University Hospital (France). All these patients complained of memory loss, for which clinical and imaging work-ups revealed no organic cause. All (except for LA, who refused this examination) were seen thereafter by a psychiatrist who confirmed the FA diagnosis. The patients’ individual medical histories are summarized in the following section and in Table 1.

##### MC

MC was a 26-year-old woman. She lived alone and had been working as a physiotherapist for 4 years when we met her. At that time, she complained of an inability to tell specific anecdotes about her life, thus impairing her social life. She had successfully completed her higher education, albeit with some difficulty, and worked in a department that she had chosen because of its routine character. She stated that she had always functioned this way, but had recently realized that it was not how normal memory should function. When she was 6 years old, she was present when one of her brothers died from a domestic accident, but nobody knows whether she was a first-hand witness of this scene. Neurological and imaging explorations were normal. MC underwent two neuropsychological examinations at our institution. A range of cognitive functions were explored, including memory, executive functions, and dissociation. An autobiographical memory exploration showed that she had difficulty giving certain details about the memories she recalled, such as their spatiotemporal context and her own actions during those events, but displayed surprising preservation of events that had happened within the previous 12 months. She had no problems with anterograde episodic memory or with other cognitive functions, although she did perform poorly on the Wisconsin Card Sorting Test (Heaton et al., 1993), suggesting poor cognitive flexibility.

##### CG

CG was a 24-year-old woman who worked as a hairdresser. She lived with her boyfriend. She had had a minor stroke 1 year before we met her. This had caused minor motor disorders, from which she recovered within the space of a few months. She had sought a consultation in a neurology department because she had lost some memories about her life (e.g., her sister’s wedding). These disorders could not be explained by the minor stroke, which affected the left sylvian area, and no other organic etiology was found, leading to the diagnosis of FA. Neuropsychological exploration revealed difficulty recalling autobiographical events especially for those encoded in the previous 12 months (since the stroke). There was no anterograde memory disorder and no other cognitive dysfunction. She had minor depressive symptoms.

##### HS

HS was a 44-year-old man. He used to be a factory worker prior to the onset of sudden and massive retrograde amnesia associated with the loss of his personal identity. He described waking up one morning and not remembering anything about himself. His partner said that he had seemed anxious in the weeks leading up to this event. She interpreted this anxiety as being linked to the anniversary of a transient ischemic attack that had occurred the year before. The deficit extended to procedural and semantic skills such as reading, writing, and lighting a barbecue. He displayed anterograde episodic memory difficulties. There were no clinical neurological symptoms or lesions visible on brain scans to explain this deficit. He was therefore diagnosed with FA and underwent reading rehabilitation with a speech therapist.

##### CC

CC was a 42-year-old man. He had sought a consultation at the neurology department because of memory disorders. These disorders had arisen 2 years before our first meeting, a few days after general anesthesia for spinal surgery. He had no medical history, except for chronic headaches since childhood. Like HS, CC had total retrograde episodic amnesia associated with partial loss of his personal identity. He did not remember some of the important events of his life (school years, wedding, etc.), but did have some knowledge about his life (e.g., military service and parents’ divorce), associated with some fleeting and imprecise perceptions. Nevertheless, because of the relative preservation of his anterograde memory, he was able to relearn some of his life events. He complained of problems in his life and at work because of his memory problems, which he managed by taking notes and with the help of his colleagues.

##### LA

LA was a 40-year-old man. He was a roofer. He complained of severe fatigue associated with a major memory deficit for events that had taken place the year before we met him. These difficulties arose after he had lost consciousness while playing video games. No neurological cause was found. This patient was in the midst of moving house after a separation. A neuropsychological exploration showed that his difficulties seemed to be more on the anterograde side than on the retrograde one, as he was able to recount personal events that had occurred in his childhood and as recently as the previous year with a great many details and no apparent difficulty.

Patients with FA did not differ significantly from FA controls on the ESR-r strategic free recall score assessing anterograde memory (*U* = 9.5, *z* = −1.46, *p* = 0.14).

#### Patients With Transient Global Amnesia

Neuropsychological examinations of the patients with TGA were conducted on their admission to the emergency department of Caen University Hospital. They all met Hodges and Warlow (1990)’s clinical criteria for TGA. All attacks were witnessed and reported by an observer, and were characterized by massive anterograde amnesia but no other cognitive disorders. After their admission, all patients underwent a neurological examination and a brain CT scan, which were normal. No patient exhibited identity loss, neurological symptoms or epileptic features, or had a history of recent head injury. All patients were seen during the acute phase of TGA and presented major memory disorders (see Table 2). All attacks resolved within 24 h. Table 2 summarizes the medical histories of these patients.

Patients with TGA (*n* = 5) had lower anterograde memory scores than either TGA controls (*U* = 0, *z* = −2.65, *p* < 0.01, *r* = 0.82) or patients with FA (*n* = 5, *U* = 0, *z* = −2.51, *p* < 0.01, *r* = 0.82). Individually, all patients with TGA had anterograde episodic memory scores below the pathological cut-off (Table 2).

### Measurements

#### Autobiographical Memory

##### Test session

In the patients with FA, EAM was assessed with a task inspired by the TEMPau task (Piolino et al., 2000) and the Autobiographical Interview (Levine et al., 2002). Patients were asked to recount autobiographical events for five lifetime periods (see Piolino et al., 2000) covering the entire lifespan and adapted to their age. Given their young age, Patients MC (26 years) and CG (24 years) were only assessed for three periods: 0–17 years, 18 years upwards except for the previous year, and previous 12 months. Participants were asked to produce four narratives for each period (except for the previous 12 months) in response to a cue (meeting, academic or work-related event, journey, or family event). For the previous 12 months, the cues were the same as in the TEMPau task (2000). Owing to the particular conditions in which it was administered to the patients with TGA, we used an abridged version of this task for them. Participants were asked to produce just one event of their choice for each of the five periods (no cue was given). Controls (FA controls and TGA controls) were administered the same abridged version. To compare the patients with FA with the patients with TGA and the control groups, scores were averaged to yield a single score per person.

General instructions, taken from the Autobiographical Interview (Levine et al., 2002), were the same for all participants:

I am going to ask you to tell me about an event that happened to you in different periods of your life. You must have been personally involved in each event and have a precise recollection of this involvement. So do not pick events that you only heard about from others. They must be events situated in a specific time and place. For example, you cannot tell me that you used to play basketball when you were at school, but you can tell me about an event that happened during a specific basketball game. I will ask you to give as many details as possible about these events, so be sure to choose events you feel comfortable discussing in detail.

After participants had recounted an event, the examiner asked, “Is that everything you can say about it?” If necessary, participants were urged to be more specific, in the same way as in the TEMPau task. Participants were given a form containing specific questions they had to answer, such as “What happened? What were your perceptions, your feelings, your thoughts? Who was present? What happened before and after the event? Where did that event take place? How old were you or what year was it when it happened? Which month or season? What time (morning, afternoon, evening)?” All narratives were transcribed verbatim. Five different raters scored these transcripts. The scoring was both qualitative and quantitative. The *qualitative* scoring included specificity and episodic re-experiencing scores (see sections “Specificity” and “Episodic Re-experiencing”), while the *quantitative* scoring involved segmenting and categorizing the narratives in order to calculate the pronouns ratio, internal details ratio, and semantic details ratio (see sections “**Internal Details Ratio”**). The *autobiographical* scores (internal details ratio, specificity, and episodic re-experiencing) and I-*self* scores (pronoun ratios) were averaged across the lifetime periods and expressed as a single score per participant.

CB (author) carried out the scoring for the entire corpus. Her scoring was compared with that of the four other scorers. PQ (corresponding author) and RL (Ph.D. student) undertook the double qualitative scoring (approx. 9% of the corpus each). AP (third-year psychology student), MG and AL (fourth-year speech therapy students) undertook the double quantitative scoring (between 6 and 9% of the corpus each).

##### Specificity

The specificity score (0-4), reflecting the uniqueness in time and place of each memory, was calculated according to the scoring rules of the TEMPAu task (Piolino et al., 2000, p. 188).

##### Episodic Re-experiencing

To assess the episodic richness and the strength with which participants conveyed a feeling of re-experiencing, we used the episodic re-experiencing score of the Autobiographical Interview (Levine et al., 2002). This is a qualitative composite rating (/18) made up of a score (/6) of overall episodic richness and four ratings (/3) for time, place, and perceptual and emotional details. There was a satisfactory level of interrater agreement. Cronbach’s α was between 0.73 and 0.80 for specificity, and between 0.75 and 0.79 for episodic re-experiencing.

##### Internal details ratio

Narratives were segmented and categorized to provide three different indicators. Our procedure served to (1) define the main event in the narrative (the most detailed event if the participant evoked different memories), and (2) segment each narrative into informational details, as in Levine et al. (2002). We added a supplementary segmentation rule as per Levine et al. (2002), in order to segment information marking discontinuities in time or changes in perspective in the narrative. To simplify the procedure and maximize interrater agreement, we decided that each proposition would only contain one verb and one subject. The only exceptions concerned words pronounced by the participant or other characters in summary form (e.g., “She told me to go to the bathroom”) or dialogue form (e.g., “I said, ‘Are you crazy?”’). Categorization of details was the same as in Levine et al. (2002). An *internal detail* had to be directly linked to the main event described by the participant and give information of an episodic nature (place, time, feeling, etc.). The *external details* category mainly contained semantic details (factual or extended events that did not require recollection of a specific time and place), as well as repetitions and other events.

Interrater reliability was assessed with Cohen’s kappa on about 10% of the narratives, with satisfactory interrater agreement on the categorization of internal/external details (0.67) and scoring categories (0.66).

We calculated the internal details ratio as in Levine et al. (2002):


(1) $$\frac{\mathrm{number}\,\mathrm{of}\,\mathrm{internal}\,\text{details}}{\mathrm{total}\,\mathrm{number}\,\mathrm{of}\,\text{details}}$$

Interrater reliability for the internal details ratio was very satisfactory (Cronbach’s α = 0.915-0.999).

#### Pronoun Use (I-Self Quantitative Assessment)

We aimed to calculate the first-person and general pronoun ratios solely for propositions corresponding to memory *per se*, and thus avoid counting the use of *I* in metacognitions (e.g., “I’m not sure I remember”). For each proposition we retained, observers placed the subject in one of the categories shown in Table 3.

**TABLE 3:** Pronoun classification.

**Category**	**Subject (pronoun)**	**Score**
First-person pronoun	I	First-person pronoun ratio
Indefinite third-person pronouns	It, somebody… It was amazing/somebody told me, etc.	General pronoun ratio
Self-references	Number of self-references per proposition: *I, me, myself*, *etc.*	Self-reference ratio
Group references	Number of group references per proposition: *we, us*, *etc.*	Group reference ratio

The ratios were calculated solely for propositions that contained a subject. The final ratios corresponded to the numbers of propositions containing first-person or general pronouns relative to the total number of propositions containing subjects.

The two pronoun ratios (first-person and general pronouns) allowed us to measure the proportions in which these pronouns were used in narratives (Table 3).

We also calculated two broader scores to measure the proportions of self-references and references to a group in the narratives. We began by counting the number of self-references in each proposition, including all references to the self, using personal (*I*), reflexive (*me*), or possessive (*my*) pronouns. We then calculated the self-reference ratio by summing all the self-references in each narrative and dividing that number by the total number of words in the narrative. The group reference ratio was calculated in the same way, by summing all the expressions where participants included themselves in a group (e.g., *my mother and I*, *we*, *us*). A specificity of French language, the undefined pronoun *on* can be used as a first-person plural pronoun in popular language, and was therefore considered as such if it was used in this way (in these cases, it was not included as an undefined pronoun in the calculation of the general pronoun ratio).

For each narrative, we calculated all the pronoun ratios and scores, and averaged them to keep one score per participant. Cohen’s kappa showed satisfactory interrater agreement on propositions (0.71), self-references (0.94), and group references (0.85).

#### Dissociation

We used the French version (Moyano et al., 2004) of the Dissociative Experiences Scale (DES; Bernstein and Putnam, 1986). This 28-item self-report scale has been validated in different clinical populations (Bernstein and Putnam, 1986), and is frequently used in research on dissociation (for a review, see McKinnon et al., 2016). During the test phase, for each of the 28 situations, participants had to indicate the frequency with which they experienced the dissociation situation that was described, on a line running from 0% (*Not at all*) to 100% (*Every time*). We calculated three scores, beginning with the total (28-item) DES score, to obtain an overall dissociation assessment. Next, we calculated *dissociative amnesia* and *depersonalization-derealization* subscores from the items identified in Bernstein and Putnam (1986). Each of these three scores was obtained by averaging scores on the corresponding items, and was expressed as a percentage. The higher the percentage, the higher the level of dissociation. Scores obtained by each patient are presented in Tables 1, 2.

### Statistical Methodology

Statistical analysis were performed with non-parametric tests. We compared EAM scores, pronoun ratios, and dissociative scores between groups (FA, TGA, controls) with the Mann-Whitney *U*-test. We also ran within-group comparisons study the use of first-person vs. general pronouns in each group of patients. These analyses were performed with the Wilcoxon signed-rank test. We conducted individual analyses, comparing the proportion of first-person vs. general pronouns for each patient using the chi-square test (χ^2^). Effect sizes were expressed by Spearman’s rho (*r*).

We then calculated Spearman correlations in each group of patients (FA and TGA), to determine the links between the three processes we were exploring. More specifically, we calculated correlations (1) between pronoun ratios and EAM indicators, (2) between EAM scores and dissociative scores; and (3) between pronoun ratios and dissociative scores.

## Results

### Autobiographical Memory

**FIGURE 1:**
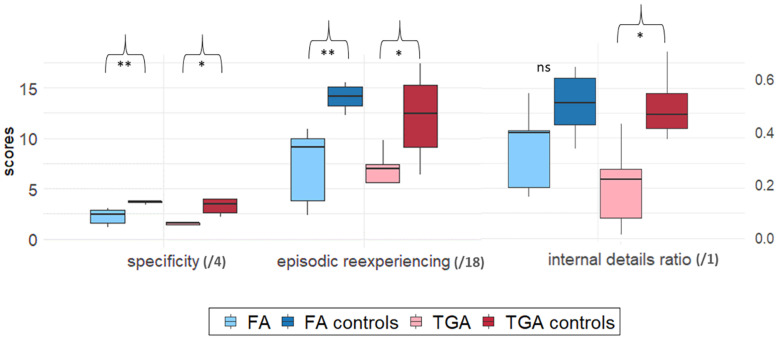
Group comparison on EAM scores. Boxplots were constructed from median and 1st and 3rd quartile scores. Bars corresponds to 1.5*IQR. Significant differences between each patient group (FA or TGA) and its matched control group (FA controls and TGA controls) are marked as follows: *p < 0.05; **p < 0.01. *ns*, non-significant.

Analysis revealed no difference between patients with FA vs. TGA on either the internal details ratio (*U* = 8, *z* = −0.84, *p* = 0.40), specificity score (*U* = 7.5, *z* = −0.94, *p* = 0.35), or episodic re-experiencing score (*U* = 9, *z* = −0.63, *p* = 0.53) (Figure 1). Patients in both groups scored lower than their matched controls on both specificity (FA: *U* = 0, *z* = −2.85, *p* < 0.01, *r* = −0.80; TGA: *U* = 2, *z* = 2.3, *p* < 0.05, *r* = −0.69) and episodic re-experiencing (FA: *U* = 0, *z* = −2.85, *p* < 0.01, *r* = −0.79; TGA: *U* = 4, *z* = −1.91, *p* = 0.05, *r* = −0.58). Patients with TGA differed significantly from their control group on the internal details ratio (*U* = 2, *z* = −2. 28, *p* < 0.05, *r* = −0.69).

Table 4 sets out patients’ individual mean scores on these three EAM indicators.

**TABLE 4:** Individual participants’ retrograde memory scores.

	**MC**	**CG**	**HS**	**CC**	**LA**	**MP**	**TC**	**CP**	**AD**	**GC**
Group	FA	FA	FA	FA	FA	TGA	TGA	TGA	TGA	TGA
Specificity (/4)	3	2.46	1.61	1.16	2.83	1.6	3	0	1.6	1.4
Episodic re-experiencing (/18)	9	9.92	3.85	2.32	10.88	5.6	9.8	0	7.4	7
Internal details ratio (/1)	0.58	0.42	0.20	0.16	0.43	0.23	0.46	0.01	0.08	0.28

### Pronoun Use (I-Self)

#### Pronoun Use in Functional Amnesia Group

**FIGURE 2:**
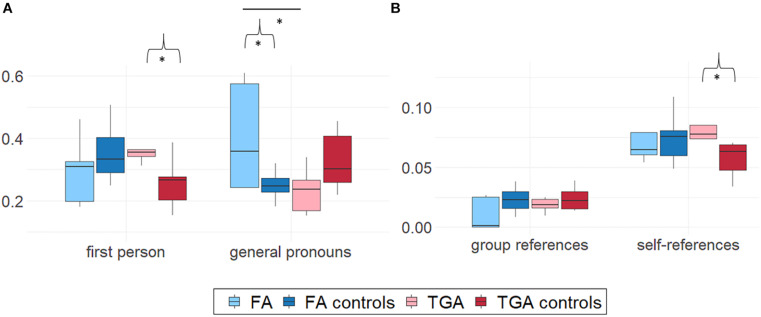
Group comparison on **(A)** pronoun ratios and **(B)** ratios of propositions containing group- or self-references in participants’ narratives. Boxplots were constructed from median and 1st and 3rd quartile scores. Bars corresponds to 1.5*IQR. Mann-Whitney *U*-tests. Brackets indicate significant differences between each patient group (FA or TGA) and its matched control group. Horizontal bars between FA and TGA or between the two control group boxplots correspond to significant differences between these groups. *p < 0.05.

Mann-Whitney *U*-tests showed that the narratives of patients with FA contained more general pronouns (*M* = 0.41, *SD* = 0.18) than those of both FA controls (*M* = 0.25, *SD* = 0.05, *U* = 4, *z* = 2.27, *p* < 0.05, *r* = 0.63) and patients with TGA (*M* = 0.23, *SD* = 0.08, *U* = 2, *z* = 2.09, *p* < 0.05, *r* = 0.66) (Figure 2A). Regarding first-person use, patients with FA did not differ from either patients with TGA (*p* = 0.14) or FA controls (*p* = 0.61).

Wilcoxon tests did not show any differences between general pronoun use and first-person pronoun use in the FA group (*p* = 0.69).

There were no differences in the use of self-references or group references between the narratives of patients with FA (self-references: *M* = 0.07, *SD* = 0.02; group references: *M* = 0.01, *SD* = 0.01) and those of either FA controls (all *ps* > 0.16) or patients with TGA (all *ps* > 0.68) (Figure 2B).

#### Pronoun Use in Transient Global Amnesia Group

By contrast, the narratives of patients with TGA contained more first-person pronouns (*M* = 0.39, *SD* = 0.10) than those of TGA controls (*M* = 0.26, *SD* = 0.08, *U* = 4, *z* = 1.92, *p = 0.*05, *r* = 0.58). They did not differ from the narratives of patients with FA (*p* = 0.14) (Figure 2A). For general pronouns, patients with TGA (*M* = 0.23, *SD* = 0.08) used fewer general pronouns than patients with FA (see section “Pronoun Use in FA Group”), but did not differ from TGA controls (*p* = 0.17).

Patients with TGA were more likely to use first-person pronouns than general pronouns (Wilcoxon’s *z* = 2.02, *p* < 0.05, *r* = 0.64).

There was no difference in group references between patients with TGA and TGA controls (*p* = 0.65; see section “Pronoun Use in TGA Group” for comparisons with FA group). The narratives of patients with TGA contained more self-references (*M* = 0.08, *SD* = 0.03) than those of TGA controls (*M* = 0.06, *SD* = 0.01, *U* = 4, *z* = 1.91, *p* = 0.05, *r* = 0.58) (Figure 2B).

#### Case Study Analyses

**FIGURE 3:**
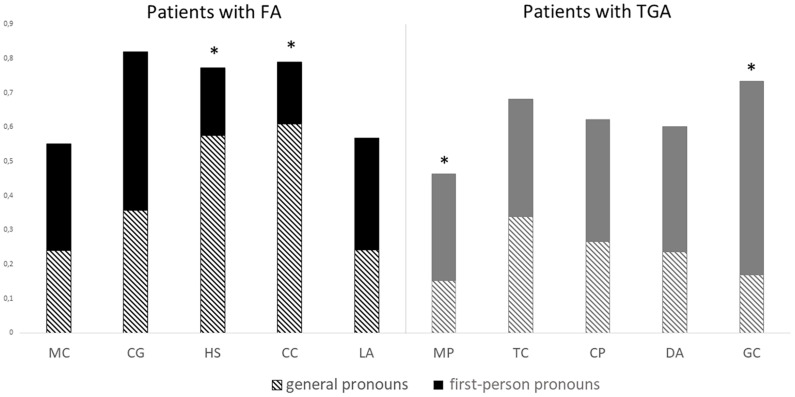
First-person pronoun and general pronoun ratios per participant. chi-square tests. *p < 0.05.

For most of the individual patients, the first-person pronoun ratio did not differ from the general pronoun ratio. In the FA group, the general pronoun ratio of patients HS (χ^2^ = 17.78, *p* < 0.001) and CC (χ^2^ = 23.4, *p* < 0.001) was higher than their first-person pronoun ratio. Two patients with TGA used first-person pronouns more than general pronouns: MP (χ^2^ = 5. 57, *p < 0.*05) and GC (χ^2^ = 20.84, *p < 0.*001) (Figure 3).

### Dissociation

**FIGURE 4:**
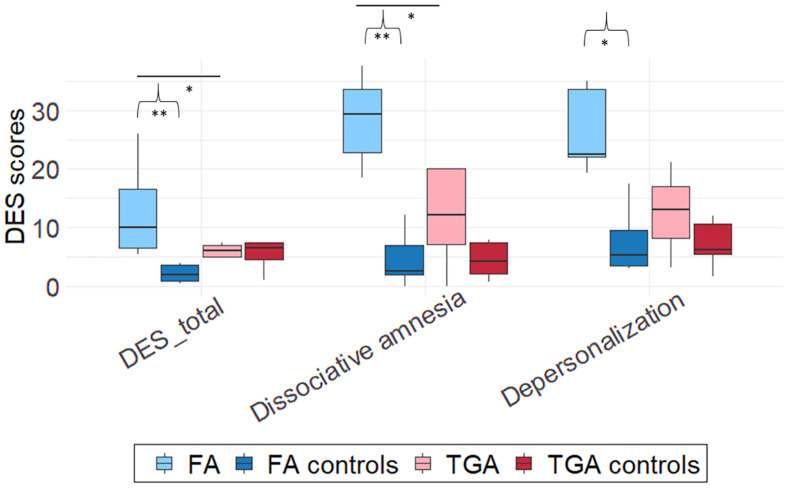
Group comparison of DES scores. Boxplots were constructed from median and 1st and 3rd quartile scores. Bars corresponds to 1.5*IQR. Significant differences between each patient group (TGA or FA) and its matched control group are marked with brackets at *p < 0.05, **p < 0.01. Mann-Whitney *U*-tests. Horizontal bars between FA and TGA or between control groups correspond to significant differences between these groups.

Mann-Whitney *U*-tests showed that the FA group had a higher total DES score (*M* = 26.4, *SD* = 7.23) than either the FA control group (*M* = 7.77, *SD* = 6.04, *U* = 0, *z* = 2.85, *p* < 0.01, *r* = 0.79) or the TGA group (*M* = 12.50, *SD* = 7.04, *U* = 1, *z* = −2.3, *p* < 0.05, *r* = 0.73) (see Figure 4).

For the *dissociative amnesia* score, patients with FA (*M* = 28.36, *SD* = 7.7) achieved significantly higher scores than either matched controls (*M* = 4.64, *SD* = 4.5, *U* = 0, *z* = 2.85, *p <* 0.01, *r* = 0.79) or patients with TGA (*M* = 11.86, *SD* = 8.6, *U* = 2, *z* = −2.09, *p* < 0.05, *r* = 0.66).

For the *depersonalization-derealization* score, patients with FA (*M* = 12.89, *SD* = 8.48) scored significantly higher than matched controls (*M* = 3.75, *SD* = 5.34, *U* = 3.5, *z* = 2.34, *p* < 0.05, *r* = 0.65). The difference between the FA and TGA (*M* = 5.1, *SD* = 3) groups was not significant (*p* = 0.14).

There were no significant differences either between the TGA group and matched controls (all *p*s > 0.41), or between the two control groups (all *p*s > 0.07).

### Correlation Analysis

#### Correlations Between Episodic Autobiographical Memory Scores and Self-Awareness

In the FA group, we found a positive correlation between episodic re-experiencing scores and first-person pronoun use (*r* = 0.90, *p* < 0.05). The more patients with FA used first-person pronouns, the more their narratives reflected episodic re-experiencing. We did not find this correlation in the TGA group. We did not find correlations between general pronoun use and EAM scores (see Table 5). There were no significant correlations between DES and EAM scores in either the FA or TGA groups.

**TABLE 5:** Correlations between EAM and self-awareness scores.

	**Specificity**		**Episodic re-experiencing**		**Internal details ratio**	
Group	FA	TGA	FA	TGA	FA	TGA
First-person pronoun	0.60	−0.46	0.90*	0.10	0.60	−0.10
General pronouns	−0.10	0.21	−0.70	0.40	−0.10	0.10
DES *dissociative amnesia*	0.10	−0.55	−0.60	−0.15	0.10	−0.67
DES *depersonali-zation*	0.40	−0.62	−0.10	−0.20	0.40	−0.30

Spearman’rho correlations. Significant correlations are indicated with bold text *p < 0.05.

#### Correlations Between Pronoun Use and Dissociative Scores

There were no correlations between the DES score and pronoun ratios, except in the TGA group, where we observed a correlation between first-person pronoun use and the DES depersonalization score (*r* = 0.90, *p* < 0.05).

To sum up, our results revealed comparable impairments on EAM in FA and TGA patients compared with their matched controls. As expected, FA patients had a higher level of dissociation compared to other groups. Regarding pronoun use, FA patients tended to use more general pronouns than the other groups while TGA patients used more first-person pronouns compared to FA patients, and also, more surprisingly, compared to healthy subjects. First-person use was correlated to EAM scores only in FA group. We found no correlation between pronoun use and DES scores.

## Discussion

### General Pronoun Use as a Marker of Structural Dissociation in Functional Amnesia

Consistent with our first hypothesis, the FA and TGA groups exhibited comparable impairments on the three EAM indicators we used (specificity, episodic re-experiencing, and internal details ratio), compared with their matched controls.

One of the main objectives of our study was to explore and compare I-self functioning through pronoun use by the two groups. We found specific patterns of pronoun use in the FA and TGA groups. Patients with FA tended to use more general pronouns than either controls or patients with TGA. In a healthy population, using the first-person pronoun in narratives, as opposed to general pronouns, is known to be a marker of truth (for a review, see Pennebaker et al., 2003). Consistent with this idea, in an analysis of five samples from different studies, Newman et al. (2003) showed that liars tend to use first-person pronouns less in their narratives, compared with persons telling the truth. More recently, a cross-cultural study confirmed this tendency for people belonging to Caucasian cultures (Taylor et al., 2017), such as the patients in our sample. According to Pennebaker et al. (2003), liars avoid first-person use and self-references because of the absence of personal experience about the narrative’s content. This situation may be comparable to that experienced by some of our patients with FA, especially those with the most severe dissociation from their past, HS and CC (see descriptions in section “Patients With FA”), as we found that their general pronoun ratio was higher than their first-person pronoun ratio. Moreover, the dissociative amnesia DES score was highest in the FA group, suggesting the presence of structural dissociation. Some dissociative symptoms may be more severe among patients with FA, and contribute to this tendency to act as a stranger to their own memories. Structural dissociation affects the structure and thence the continuity of the self (Steele et al., 2009). The present self is separated (dissociated) from former selves, thus disturbing the recall of events that occurred before this separation. This tendency may be explained by a protective mechanism whereby the focus is on the present. There are many reasons for distancing oneself from the past, notably to preserve self-esteem when it is threatened by traumatic memories. Even in typical individuals, this may be the case for memories of personal or professional failures (for a review, see Wilson and Ross, 2003). Fuchs (2007) suggested that in patients with borderline personality disorder, the disconnection of the self from the past and/or future may serve as a defensive mechanism to avoid incoherencies. In FA, structural dissociation symptoms may be linked to the blocking of unwanted memories, reminiscent of the Freudian concept of repression. In some recent conceptions, this kind of mechanism has been linked to the functioning of the frontal lobe (Anderson and Green, 2001; Harrison et al., 2017). From this perspective, there are at least two different theories about FA involving frontal functions: one suggesting a disturbance of frontal, hippocampal and amygdalar activity following hyperarousal linked to stress factors (Staniloiu et al., 2010; Bidzan, 2017), triggering an *amnesic block syndrome*; the other suggesting an increase in frontal lobe activation linked to an active mechanism for suppressing relevant memories (Kopelman, 2000; Anderson and Green, 2001; Harrison et al., 2017).

One patient with FA (LA) presented an atypical profile, with no difficulties on the retrograde side (rich narratives with many episodic details) but considerable anterograde amnesia, confirmed by anterograde memory scores. This patient’s amnesia had arisen in a context of family problems (see section “Patients With FA” for a description). Most of LA’s narratives had a positive valence and contrasted strongly with his description of his current life. In the rare cases of anterograde functional amnesia reported in scientific publications, psychogenic factors concern difficulty facing up to the future owing to the onset of an event perceived of as difficult (Staniloiu et al., 2018). According to Staniloiu et al. (2018), in these patients, the past may be embellished and idealized, explaining the preservation of the retrograde side of the autobiographical memory (i.e., Patient L described in Staniloiu et al., 2018). Thus, structural dissociation may occur when individuals have an interest in dissociating themselves from other parts of their cognition, explaining the links with a defensive process. This break in accessibility may not be specific to the retrieval of past memories, and may equally affect anterograde or even future projection components, with the rejection of an unwanted present or future by means of the same mechanisms as for a dissociated past. The hypothesis of a break in accessibility is coherent with the reported efficacy of hypnotherapy for these kinds of symptoms (Iglesias and Iglesias, 2009; McKay and Kopelman, 2009; Deeley, 2016). Hypnosis may overcome the blocking mechanisms, and the content of inaccessible functions or memories may thus prove to have been preserved.

Most of the patients with FA (see Table 1) in our study exhibited little or no anterograde disturbance, in contrast to the disproportionate retrograde deficit. Nevertheless, most of them, except CG, complained about the encoding of new information, suggesting subtle anterograde episodic memory dysfunctions. Furthermore, these patients had a higher level of overall dissociation, and not just for dissociative amnesia items, compared with controls and patients with TGA. This suggests that different kinds of dissociation can occur concomitantly, as shown in many pathologies, such as PTSD or schizophrenia, explaining why numerous conceptions argue for a continuity between these two kind of symptoms (Holmes et al., 2005). The concomitant presence of different dissociative symptoms in patients with FA could explain the lack of correlation in our study between DES scores and pronoun use, challenging the idea that the latter is a reliable general indicator of dissociation in narratives.

### Predilection for First-Person Use Among Patients With Transient Global Amnesia: A Consequence of the Episodic Memory Failure?

Although the TGA and FA groups exhibited comparable EAM disruption, they had contrasting pronoun use profiles. Our results showed that the patients with TGA used more first-person pronouns and made more self-references than healthy participants. According to Tausczik and Pennebaker (2010), pronoun use and verb tense in narratives can tell us about the individual’s attentional focus, and we can therefore deduce that the patients with TGA had a high level of self-focus.

This result is consistent with a previous study in which we found, by using a self-awareness questionnaire, that TGA patients tended to be more self-centered in the acute phase than during recovery or several months later (Becquet et al., 2021). Regarding the first-person use in patients with other causes of episodic amnesia, Small et al. (1998) found that patients with Alzheimer’s disease tended to use more often first-person pronouns than a control group composed of caregivers. Then, the disruption of episodic memory, by impairing the patients’ ability to travel in subjective time, could be associated to an increased focus on the self and on the present. Nevertheless, in another study, Fazio and Mitchell (2009) showed a comparable first-person use between Alzheimer disease patients and healthy subjects. Further studies are needed to clarify this issue.

On the other hand, in our study, the absence of a positive correlation between first-person use and EAM scores in patients with TGA, in contrast to those with FA, suggests that this self-focus is not directly associated to episodic amnesia *per se*, but perhaps instead to psychopathological mechanisms. It is known that the TGA situation affects patients’ psychological state. During the acute phase of TGA, patients tend to be highly anxious (Inzitari et al., 1997; Pantoni et al., 2000; Noël et al., 2008). This state of anxiety arising from the TGA situation could have an impact on their narration.

Indeed, for patients in the acute phase, the environment is presumably highly threatening, and because of their anterograde deficit, they may find the general context of the environment quite impenetrable. For example, they often do not know how they arrived in the emergency room. Many of the patients’ iterative questions concern this issue, which gives rise to intense anxiety (Quinette et al., 2006). In this situation, self-focused attention may be a protective mechanism, thus explaining the exaggerated use of the first person in the narratives. Döhring et al. (2014) put in evidence, in TGA patients in the acute stage a predilection for emotion centered coping strategies, which may result from this temporary self-focused attention. Thus, it is difficult for patients in the acute phase to use action coping strategies such as planning, as they are unable to project themselves into their own future (Juskenaite et al., 2014). In this sense, because of the temporal discontinuity caused by TGA, all patients with TGA experience a kind of dissociative amnesia during the acute phase. According to Howell (2003), narcissism can be understood as a consequence of dissociation. To maintain a version of the environment that rejects the failure to understand the situation, individuals have to invest the self strongly, in opposition to the external, threatening environment or people. Contrasting with our general hypothesis, in some cases, dissociation may therefore correspond to an intense focus on the self, rather than a withdrawal, possibly explaining the absence of a link between dissociation and pronoun use in our study. This paradox could be explained by the major gaps between the different concepts that come under the heading of dissociation, which still requires definition and clinical description (Holmes et al., 2005; Steele et al., 2009).

### Transient Global Amnesia: A Dissociative Syndrome?

Finally, we examined the dissociative profile of patients with TGA, which had never previously been investigated, even though the origin of this disturbance remains a puzzle. Despite a massive disorder affecting both anterograde and retrograde episodic memory, and despite the fact that the TGA situation could correspond, *a posteriori*, to a situation of dissociative amnesia, the patients with acute TGA studied here had a very different dissociative profile from that of the patients with FA. When the patients with TGA were interviewed during the neurological and neuropsychological examinations, they had no difficulty telling their personal history (name, occupation, children, etc.). This preservation of the autobiographical memory, despite an episodic impairment of retrograde memory similar to that of patients with FA, was consistent with previous reports (Quinette et al., 2006; Bartsch and Deuschl, 2010; Harrison et al., 2017). Preserved identity in patients with TGA could be due to their preserved personal semantic memory (Guillery-Girard et al., 2006), which can be disturbed in FA (Staniloiu et al., 2010). This aspect was not formally investigated in our study. Personal semantic preservation could allow patients with TGA to maintain the continuity between their current self and the self before the onset of the amnesia. This suggests that the sense of continuity of the self is not solely an episodic memory function, as argued by studies showing that non-episodic future projections are possible in TGA (Juskenaite et al., 2014). Studies among other amnesic populations also argue in favor of this suggestion, by showing that the sense of self is preserved in Alzheimer’s disease (Klein et al., 2003; Caddell and Clare, 2010; Eustache et al., 2013), despite the polysemy of the term *self* that can lead to contradictory conclusions (for a review, see Caddell and Clare, 2010).

## Limitations and Conclusion

The main limitation of the present study stems from the scarcity of functional amnesic patients and the variability of their neuropsychological profiles that make the study of a large and homogeneous cohort almost impossible. We thus chose a methodology based on a comprehensive assessment of the autobiographical narratives, in order to find specific indicators of I-self functioning which implies to consider the narratives of the event without external details. Such a methodology would be time consuming in large samples. Nevertheless, our methods allowed us to explore different patterns of links between EAM, pronoun use, and dissociation in patients with FA and patients with TGA presenting EAM disorder from different etiologies. Although the two groups had similarly impaired EAM, the patients with FA had high levels of dissociation and tended to use general pronouns in their narratives. By contrast, the patients with TGA did not exhibit dissociation in the classic assessment, and tended to use a higher proportion of first-person pronouns in their narratives than healthy controls did. Both these amnesic syndromes can sometimes be linked to psychogenic factors and have a mysterious psychogenesis. Nevertheless, despite these similarities, the two conditions seem to involve different networks in the brain. In addition, there were disparities among patients in the same group. These disparities could be explored by considering the multiplicity of medical histories and precipitating events leading to FA and TGA. For example, some patients with TGA report precipitating events classified as *psychological*, whereas others report physical precipitating events (Quinette et al., 2006). In our study, we showed that profiles can be differentiated by exploring patients’ narratives. This methodology has numerous advantages in behavioral studies, not least avoiding long test sessions for patients who are liable to tire during examinations. Moreover, it allows data to be collected from a natural perspective without the filter of self-reports, which can yield questionable results when they are used in patients with self-awareness disorders.

## Data Avilability Statement

The raw data supporting the conclusions of this article will be made available by the authors, without undue reservation.

## Ethics Statement

The studies involving human participants were reviewed and approved by Local Ethics Committee of the University of Caen. The patients/participants were given an information form about the research and provided their oral consent to participate in this study.

## Author Contributions

CB and PQ: study conception and design. CB, JC, PL, and FV: acquisition of data. CB, JD, FV, FE, and PQ: analysis and interpretation of data. CB: drafting of manuscript. JD, FV, FE, and PQ: critical revision. FE and PQ: supervision of the project. All authors contributed to the article and approved the submitted version.

## Conflict of Interest

The authors declare that the research was conducted in the absence of any commercial or financial relationships that could be construed as a potential conflict of interest.

## Publisher’s Note

All claims expressed in this article are solely those of the authors and do not necessarily represent those of their affiliated organizations, or those of the publisher, the editors and the reviewers. Any product that may be evaluated in this article, or claim that may be made by its manufacturer, is not guaranteed or endorsed by the publisher.

## Footnotes

1 The term *dissociative amnesia* can refer to either a type of dissociative symptom or a clinical entity described in the DSM-V. Here, to disambiguate its use, we used *functional amnesia* (FA) to designate the clinical entity, and *dissociative amnesia symptoms* to designate the symptoms.
